# Supplementary material for: Comprehensive analysis of intercellular communication in the thermogenic adipose niche
Source: Commun Biol. 2023 Jul 21;6:761. doi: 10.1038/s42003-023-05140-2 (PMC10361964; doi:10.1038/s42003-023-05140-2)
Supplement: Supplementary file 2 — Supplementary Information [file 42003_2023_5140_MOESM2_ESM.pdf]

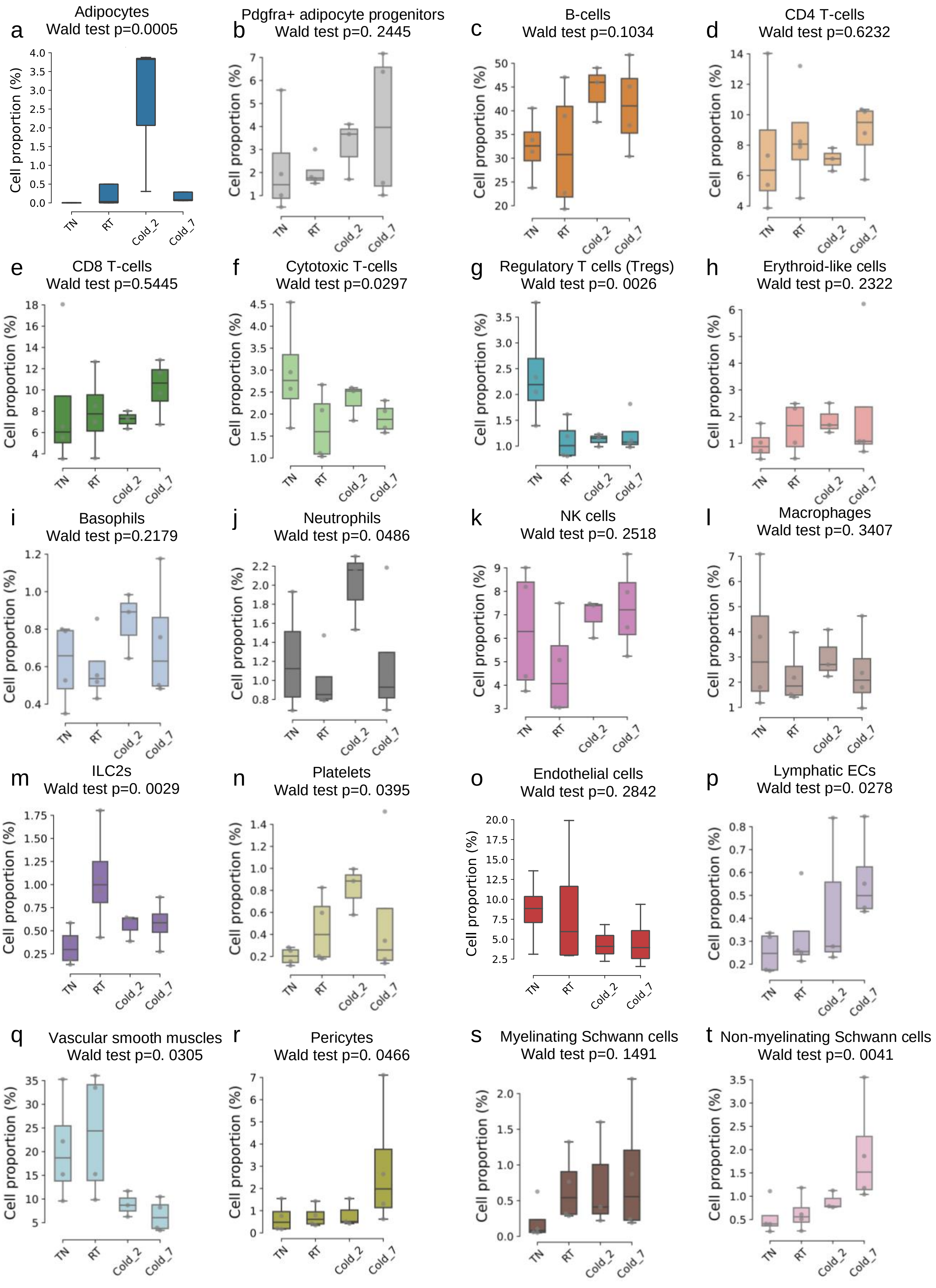
a
Adipocytes
Wald test p=0.0005
b
Pdgfra+ adipocyte progenitors
Wald test p=0. 2445
c
B-cells
Wald test p=0.1034
d
CD4 T-cells
Wald test p=0.6232
e
CD8 T-cells
Wald test p=0.5445
f
Cytotoxic T-cells
Wald test p=0.0297
g
Regulatory T cells (Tregs)
Wald test p=0. 0026
h
Erythroid-like cells
Wald test p=0. 2322
i
Basophils
Wald test p=0.2179
j
Neutrophils
Wald test p=0. 0486
k
NK cells
Wald test p=0. 2518
l
Macrophages
Wald test p=0. 3407
m
ILC2s
Wald test p=0. 0029
n
Platelets
Wald test p=0. 0395
o
Endothelial cells
Wald test p=0. 2842
p
Lymphatic ECs
Wald test p=0. 0278
q
Vascular smooth muscles
Wald test p=0. 0305
r
Pericytes
Wald test p=0. 0466
s
Myelinating Schwann cells
Wald test p=0. 1491
t
Non-myelinating Schwann cells
Wald test p=0. 0041
Cell proportion (%)
TN
RT
Cold_2
Cold_7

Supplementary Figure 1. Ambient temperature changes the cellular composition of BAT, related to Figure 1. (a-t) The proportion of cells assigned to each cluster at different housing conditions in the scRNA-seq dataset of mouse BAT-SVF.

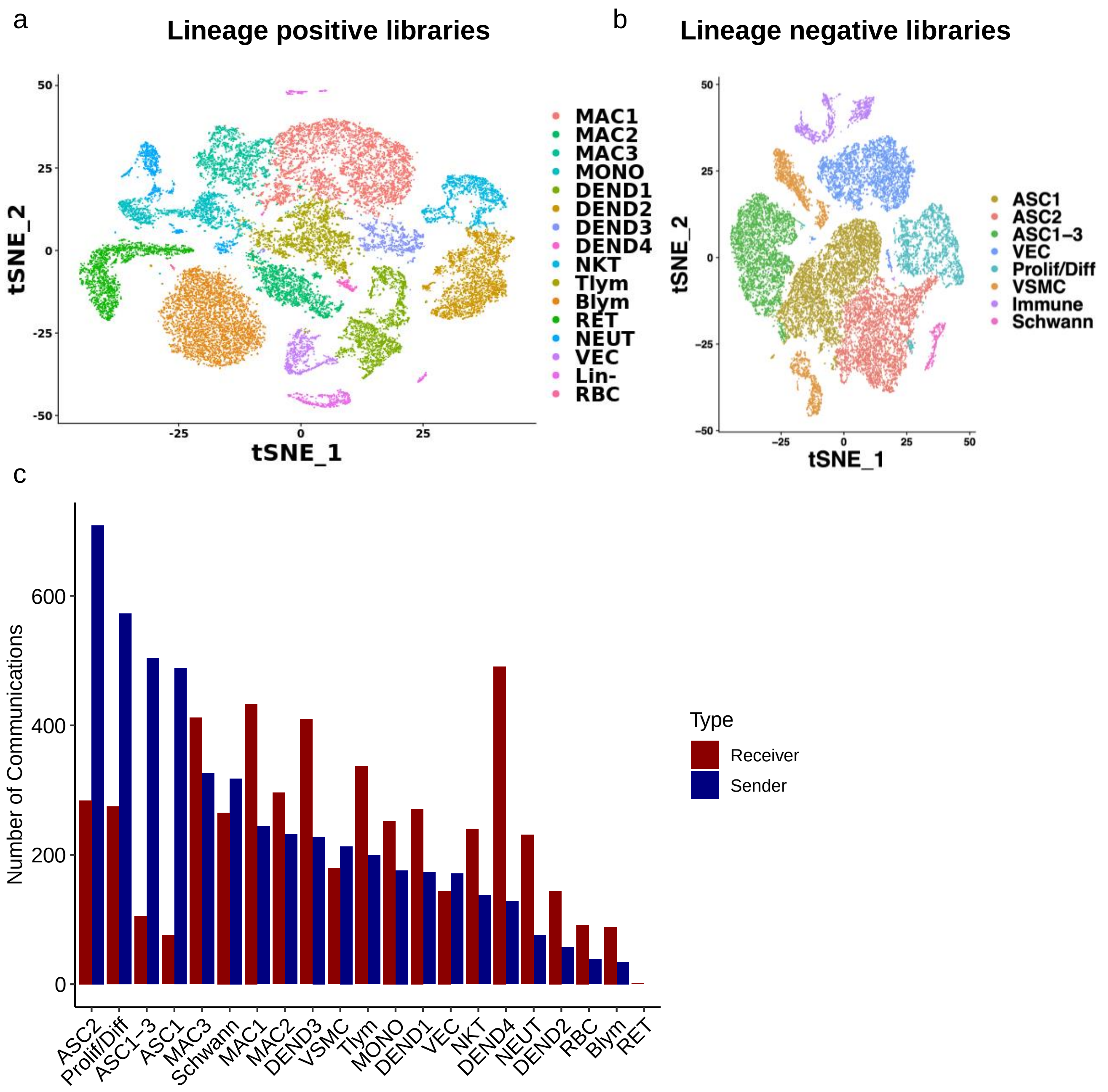

Supplementary Figure 2. Analysis of ligand-receptor interactions in single-cell transcriptomics dataset of mouse BAT[1], related to Figure 1. Unsupervised clustering of (a) lineage positive and (b) lineage negative cells from the BAT-SVF of mice housed at RT or Cold (5 °C, 4 days) represented on a tSNE map. (c) The number of significant communications involving each cell type as “sender” or “receiver”.

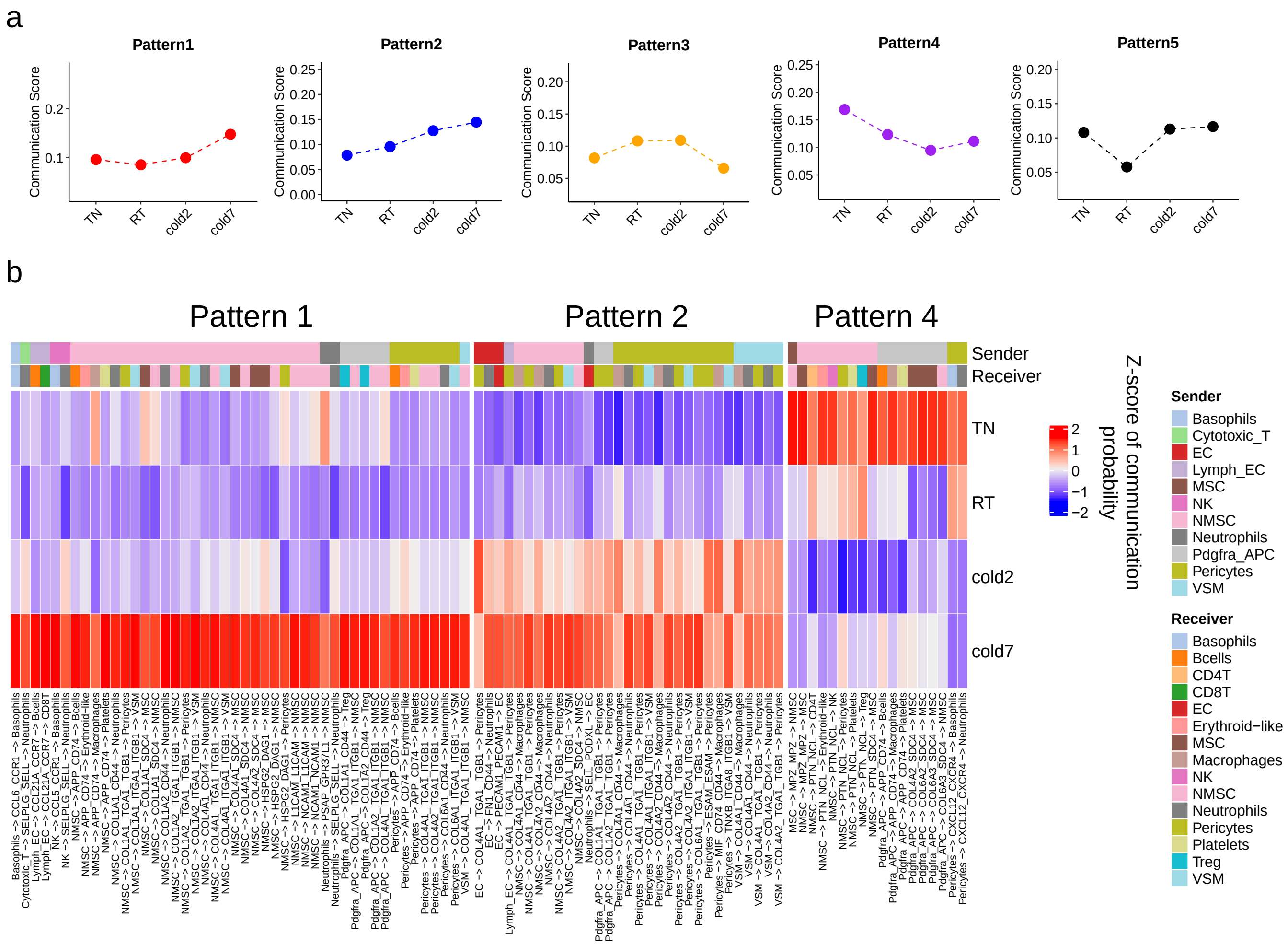

Supplementary Figure 3. Ambient temperature remodels intercellular crosstalk in BAT, related to Figure 2. (a) Classification of the predicted communications in BAT based on their pattern similarity across different conditions**.** (b) Heat map showing the communication scores for the top ligand-receptor interactions following patterns 1, 2, and 4 across different conditions.

Supplementary References

1 Burl, R. B., Rondini, E. A., Wei, H., Pique-Regi, R. & Granneman, J. G. Deconstructing cold-induced brown adipocyte neogenesis in mice. *Elife* **11** (2022). https://doi.org:10.7554/eLife.80167
