# Supplementary material for: Comprehensive analysis of intercellular communication in the thermogenic adipose niche
Source: Commun Biol. 2023 Jul 21;6:761. doi: 10.1038/s42003-023-05140-2 (PMC10361964; doi:10.1038/s42003-023-05140-2)
Supplement: Supplementary file 3 — Description of Supplementary Materials [file 42003_2023_5140_MOESM3_ESM.docx]

**Description of Additional Supplementary Files**

**File name:** Supplementary Data 1

**Description:** specifically expressed genes across 20 cell types identified from scRNAseq dataset
